# A case of woolly hair nevus, multiple linear pigmentation, and epidermal nevi with somatic *HRAS* p.G12S mutation

**DOI:** 10.1111/pde.13783

**Published:** 2019-03-12

**Authors:** Katsuhiko Nishihara, Mikiko Tohyama, Akiharu Kubo

**Affiliations:** ^1^ Department of Dermatology Ehime University Graduate School of Medicine Toon Ehime Japan; ^2^ Department of Biochemistry Keio University School of Medicine Tokyo Japan

## Abstract

Woolly hair nevus is a rare syndrome that presents as woolly hair in restricted areas of the scalp and may be associated with pigmented macules or epidermal nevus on the body. Here, we report a case of woolly hair nevus, linear pigmentation, and multiple epidermal nevi with a somatic *HRAS* c.34G>A(p.G12S) mutation.

## 1 CASE REPORT

**Figure 1:**
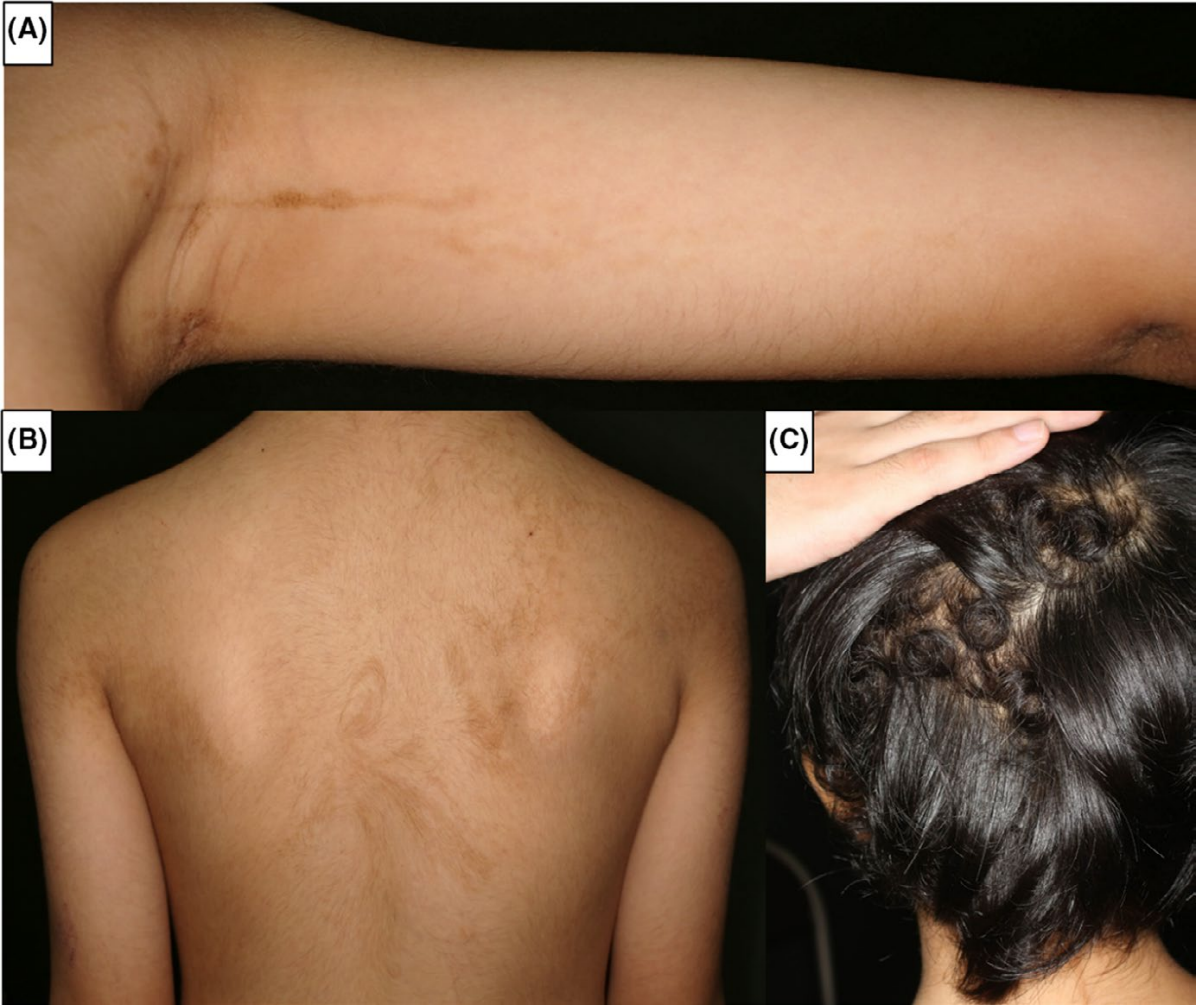
A, Clinical features. Note the linear brown verrucous papules on the left forearm. B, Linear and whorled hyperpigmentation on the back. C, Restricted woolly hair on the brown‐colored scalp

**Figure 2:**
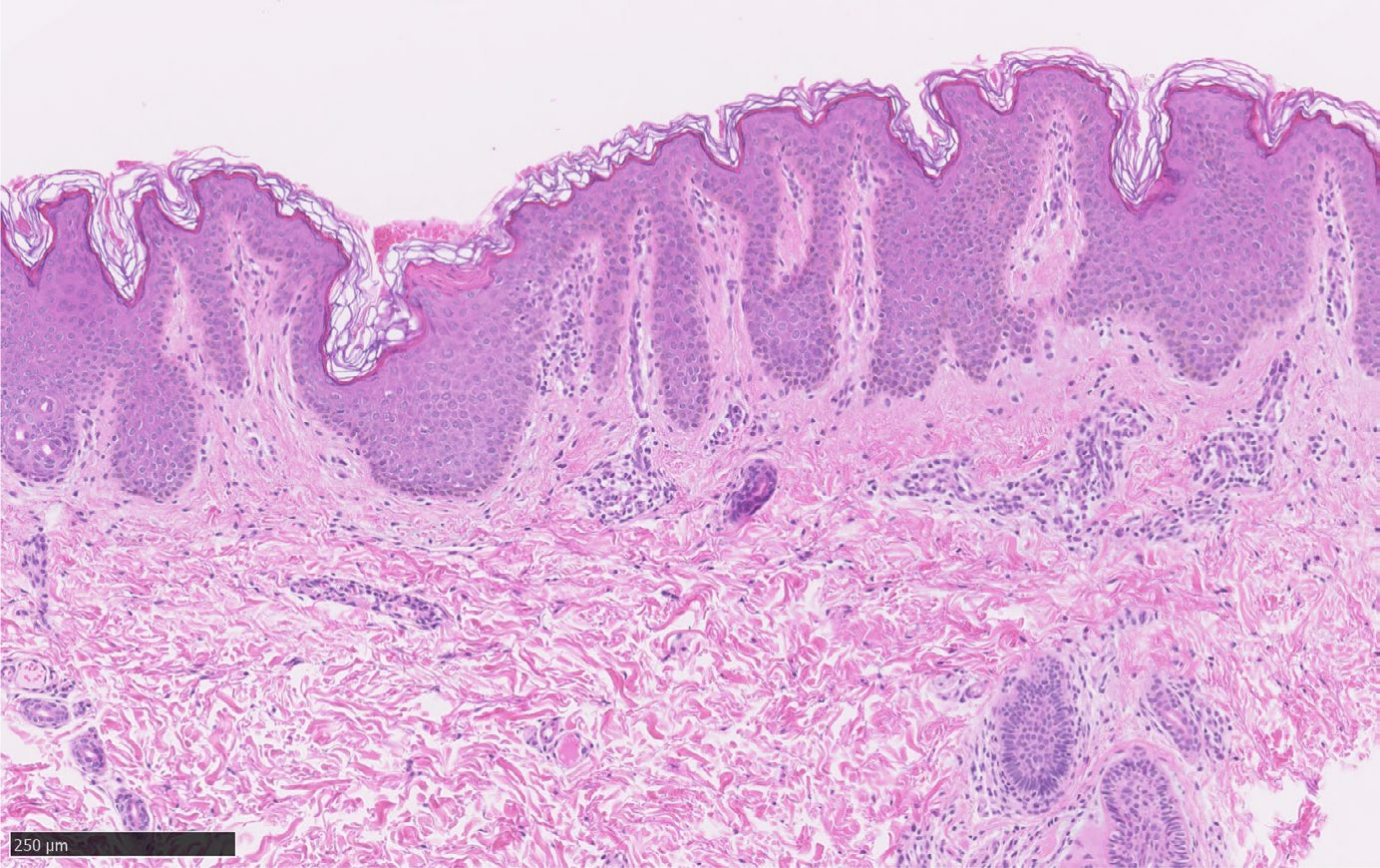
Skin biopsy histopathology. Hyperkeratosis, hypermelanosis, acanthosis, and papillomatosis of the epidermis were observed

**Figure 3:**
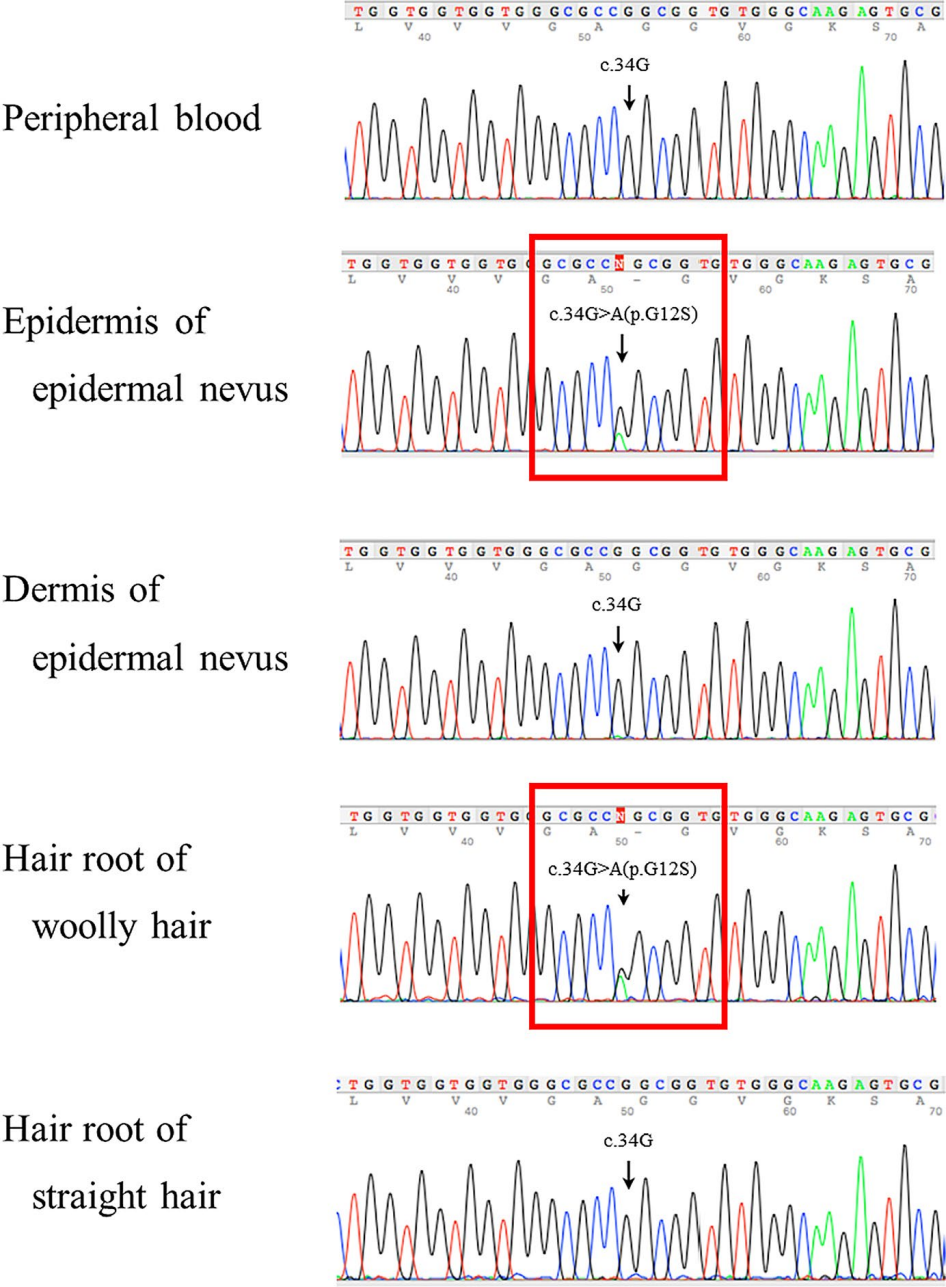
Sanger sequencing chromatogram of the indicated samples showing a heterozygous. *HRAS* c.34G>A (p.G12S) mutation specifically in the woolly hair root and the epidermis from an epidermal nevus

A 5‐year‐old Japanese boy presented to the dermatology department with multiple linear pigmentations on the arms and trunk. Linear brown verrucous papules on the left forearm were noticed at about 9 months after birth (Figure 1A), and nuchal pigment macules were recognized at 1 year old. Linear and whorled hyperpigmentation then appeared gradually on the patient's back, axilla, and chest (Figure 1B). There was no hyperkeratosis of the palms or soles. Woolly hair was observed locally on the scalp (Figure 1C). Other than the skin manifestations, the patient did not exhibit any other symptoms. Neither the patient's parents nor his sister had woolly hair or linear pigmentation. Histopathologically, a skin biopsy specimen on the axilla with linear pigmentation showed hyperkeratosis, hypermelanosis, acanthosis, and papillomatosis of the epidermis that was consistent with an epidermal nevus (Figure 2). Genetic screening was conducted after obtaining written informed consent. Next‐generation sequencing of the genomic DNA purified from the epidermis of the epidermal nevus enzymatically separated from the dermis using custom‐targeted exome sequencing panels of the Haloplex target enrichment system.^1^ Sanger sequencing revealed an *HRAS* c.34G>A (p.G12S) mutation specifically in the epidermis but not in the dermis of the epidermal nevus and the blood (Figure 3). Analyses of the mRNA determined the identical *HRAS* mutation in the hair roots of the woolly hair but not of the straight hair (Figure 3). Echocardiography and abdominal ultrasound performed after genetic diagnosis showed no abnormal findings.

## 2 DISCUSSION

The occurrence of woolly hair nevus does not differ between sexes and usually appears within the first 2 years of life.^2^, ^3^ An association between pigmented macules, an epidermal nevus, or an ectopic mongolian spot with woolly hair nevus has been reported.^2^ In previous reports of woolly hair nevus, we found 10 cases associated with linear epidermal nevi. Genetic screening was performed in three patients, who all had HRAS p.G12S mosaic mutation, similar to our case.^4^, ^5^

*HRAS* encodes an oncogenic RAS protein, mutations of which cause various skin diseases. Hafner et al demonstrated that 26 of 72 cases of epidermal nevus had *HRAS* mutations, the major ones of which result in p.G13R, not p.G12S substitution.^6^ *HRAS* mutations were also observed in 62 of 65 and in 30 of 36 sebaceous nevus cases.^7^, ^8^ Almost all mutations result in p.G13R, not p.G12S substitutions.^7^ A heterozygous HRAS p.G12S mutation was demonstrated in the epidermal nevi of our case. The epidermal nevi were slightly elevated and located in areas of rubbing contact, such as the axilla and hands. Serial lesions on the arms and trunk looked like pigmented macules or linear pigmentations. Therefore, the HRAS p.G12S mutation may cause mild verrucous hyperplasia compared to the HRAS p.G13R mutation.

Costello syndrome is a disease that produces various complications and is also caused by *HRAS* mutations.^9^, ^10^ Costello syndrome patients show growth and developmental delays, enlargement‐type cardiomyopathy, and frame abnormalities with dermatosis of woolly hair of the whole head, palmoplantar hyperkeratosis, papilloma, and acanthosis nigricans.^11^ Ninety percent of Costello syndrome patients showed HRAS p.G12S mutations.^10^ According to a report by Bertola et al.^12^ woolly hair was found in 88% of cases with HRAS p.G12S mutations and in 33% of cases with other mutation. Together, these results imply that the HRAS p.G12S mutation may play a significant role in the occurrence of woolly hair.

Fifteen percent of Costello syndrome patients also develop malignant tumors.^13^ The most common tumor is rhabdomyosarcoma, mainly involving abdomen, pelvis, and urogenital area.^13^ In addition, neuroblastoma and bladder carcinoma have occurred in several patients.^14^ Rhabdomyosarcoma and neuroblastoma develop at a median age of 2.3 years and 1.4 years, respectively.^14^ These ages at the time of diagnosis are comparable with the ages expected for sporadic cancers. On the other hand, the median age at diagnosis of bladder carcinoma in 4 cases with Costello syndrome was 13.5 years, although bladder carcinoma most frequently occurs in the elderly.^14^ If epidermal nevus patients have the mosaic *HRAS* mutation not only in epidermal nevi but also in other tissues, they may be at risk of developing neoplasms similar to those found in Costello syndrome. One patient with epidermal nevi and HRAS p.G12S mosaic mutation was reported to develop urothelial cancer at 19 years old.^15^ Therefore, our patient began annual checks for malignancies at 7 years old by abdominal/pelvic ultrasonography and by urinalysis for hematuria, which is a proposed tumor screening protocol for Costello syndrome.^13^

In conclusion, woolly hair associated with epidermal nevi may be caused by a HRAS p.G12S mosaic mutation. If a HRAS p.G12S mutation is defined, periodical follow‐up is necessary to account for the risk of carcinogenesis from early childhood. Early genetic diagnosis may be important in these patients.
